# Stiff Person Syndrome: A Rare Neurological Disorder, Heterogeneous in Clinical Presentation and Not Easy to Treat

**DOI:** 10.1155/2015/278065

**Published:** 2015-05-27

**Authors:** Susanne Buechner, Igor Florio, Loredana Capone

**Affiliations:** Department of Neurology, Regional General Hospital, Lorenz Boehler Street 5, 39100 Bolzano, Italy

## Abstract

*Background*. Stiff person syndrome (SPS) is a rare neurological disorder characterized by progressive rigidity of axial and limb muscles associated with painful spasms. SPS can be classified into classic SPS, paraneoplastic SPS, and SPS variants. Its underlying pathogenesis is probably autoimmune, as in most cases antibodies against glutamic acid decarboxylase (GAD) are observed. Similarly, paraneoplastic SPS is usually linked to anti-amphiphysin antibodies. Treatment is based on drugs enhancing gamma-aminobutyric acid (GABA) transmission and immunomodulatory agents. *Case Series*. Patient 1 is a 45-year-old male affected by the classic SPS, Patient 2 is a 73-year-old male affected by paraneoplastic SPS, and Patient 3 is a 68-year-old male affected by the stiff limb syndrome, a SPS variant where symptoms are confined to the limbs. Symptoms, diagnostic findings, and clinical course were extremely variable in the three patients, and treatment was often unsatisfactory and not well tolerated, thus reducing patient compliance. Clinical manifestations also included some unusual features such as recurrent vomiting and progressive dysarthria. *Conclusions*. SPS is a rare disorder that causes significant disability. Because of its extensive clinical variability, a multitask and personalized treatment is indicated. A clearer understanding of uncommon clinical features and better-tolerated therapeutic strategies are still needed.

## 1. Introduction

Stiff person syndrome (SPS) is a rare neurological disorder, which classically is characterized by persistent skeletal muscular stiffness [1, 2]. Coactivation of agonist and antagonist muscles mainly of the paraspinal and abdominal muscles provokes axial rigidity with lumbar hyperlordosis and postural instability. Progression of stiffness to limbs makes walking difficult or even impossible. The rigidity is accompanied by intermittent muscular cramps and spasms, potentially painful and disabling, which are often triggered by external stimuli (e.g., unexpected noise or tactile stimulation) or emotional stress. Psychiatric symptoms such as depression and generalized anxiety disorder with phobias and panic attacks are common. Sometimes psychiatric symptoms are prominent, leading to misdiagnosis of psychiatric illnesses [3]. SPS can be classified into classic SPS, paraneoplastic SPS, and SPS variants (namely, Focal or Segmental SPS, Jerky SPS, Progressive encephalomyelitis with rigidity and myoclonus, and SPS plus) [2]. SPS seems to have an autoimmune basis although its exact pathogenic mechanism is still cloudy [1, 2]. Most SPS cases are associated with antibodies against glutamic acid decarboxylase (GAD). Antibodies against the antigens gephyrin, the glycine-*α*1 receptor, or the gamma-aminobutyric acid (GABA) receptor-associated protein can also be observed. Furthermore, it is common that patients with classic SPS suffer from other autoimmune disorders such as type I insulin-dependent diabetes mellitus (DM) or Hashimoto's thyroiditis. In contrast, paraneoplastic SPS is usually linked to anti-amphiphysin antibodies and associated with malignant tumors of the breast, colon, lung, thymus, and lymphoma. The diagnosis of SPS is mainly based on the Dalakas criteria [4] that, together with the clinical features, include electromyography (EMG) findings showing continuous motor unit activity (CMUA) at rest and laboratory serology tests positive for the above-mentioned antibodies. Therapeutic strategies for SPS encompass a combination of symptomatic and immunomodulatory therapy [1, 2]. While the former consists mainly of drugs that enhance the GABA activity, the latter is aimed at altering the autoimmune attack. In particular, benzodiazepines, baclofen, some antiepileptic drugs, or intramuscular botulinum toxin are mainly used as muscle relaxants and for treatment of spasticity. Drugs that modulate the immune process include intravenous immunoglobulin (IVIG), plasma exchange (PE), rituximab, or other immunomodulatory agents (e.g., prednisone). In the case of paraneoplastic SPS, treatment of the underlying cancer is essential [5]. Finally, the disabling psychological and psychiatric features have to be managed, too, and physiotherapy is often necessary. SPS prognosis is unforeseeable and variable, also because clinical progression depends on numerous factors, such as associated autoimmune disorders or malignancy. Here we describe three cases of SPS in order to evidence the differences within the variants of SPS, to emphasize some unusual clinical features, and to demonstrate that treatment of SPS can be very difficult, even though it follows guidelines and recommendations [1].

## 2. Case Series

### 2.1. Patient 1

**Figure 1:**
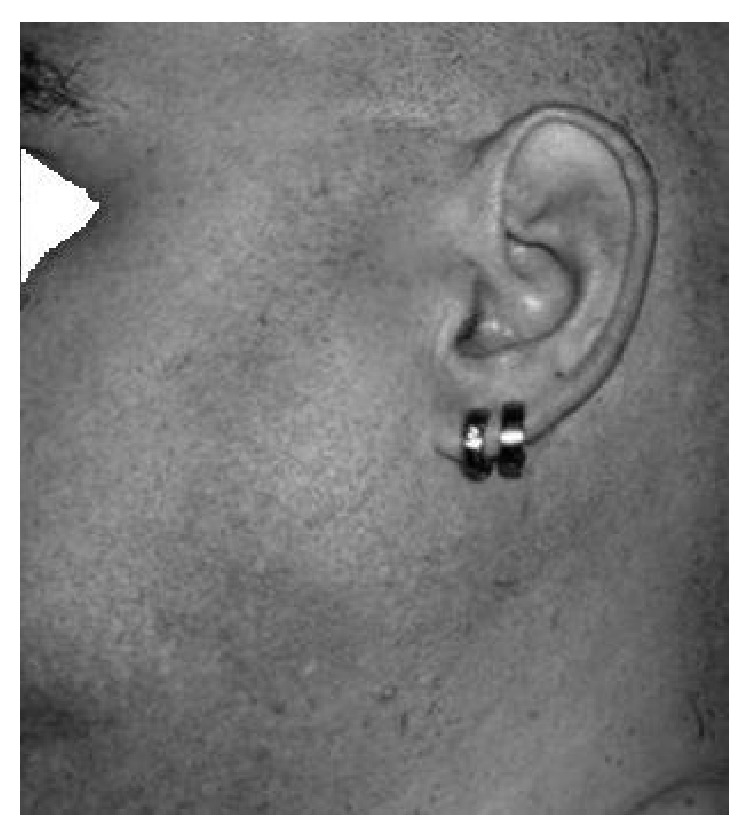
Hypertrophy of masseter muscle. Facial stiffness makes eating difficult.

Patient 1 is a 45-year-old Caucasian male affected by the classic anti-GAD-antibody related SPS. At the age of 36 the patient started complaining about pain and stiffness in proximal and axial muscles at rest. During exercise he suffered from fatigue and weakness despite the apparent presence of muscular hypertrophy and afterwards from delayed muscle relaxation and myokymia. Furthermore he reported diffuse painful muscle cramps. In the following years he exhibited progressive walking difficulties, and stiffness extended to pulmonary and facial muscles (Figure 1), leading to respiration and eating problems. Cold temperatures induced generalized muscle spasms provoking recurrent episodes of respiratory insufficiency. This disabling medical condition, added to the patient's previous health problems (type I DM from the age of 12, shoulder joint degeneration, gastroesophageal reflux, and hepatic steatosis), aggravated his anxious-depressive syndrome leading to agoraphobia with panic attacks and insomnia. EMG testing showed an axonal sensory-motor polyneuropathy, probably DM-related, and CMUA at rest. Laboratory investigations were positive for anti-GAD-antibodies (<500 KUI/l compared to a reference value of <10 KUI/l). Until today, the patient is treated with high doses of benzodiazepines, gabapentin, carbamazepine, and regularly scheduled sessions of IVIG (initially 0.4 g/kg/day given over 5 days every 2-3 months, recently 0.2 g/kg/day given over 3 days every month). At the beginning of treatment muscular symptoms improved dramatically, but progressively IVIG response has become fluctuating. The continuous need to increase benzodiazepine and gabapentin doses has led to intolerable side effects such as excessive daytime sleepiness, unsteadiness, and confusion. Progressively the patient's compliance has decreased leading to irregular IVIG infusions and uncontrolled modifications of oral therapy without doctor approval. Attempts with PE had to be interrupted due to thrombotic events.

### 2.2. Patient 2

Patient 2 is a 73-year-old Caucasian male affected by paraneoplastic SPS. At the age of 72 the patient started complaining about diffuse itching, followed by severe pain, stiffness, and oedema of his left foot. Pain and stiffness extended quickly to both legs accompanied by very painful muscle cramps that led the patient to become wheelchair-bound. EMG testing showed a left peroneal nerve lesion, probably due to significant weight reduction, and CMUA at rest. Laboratory investigations were positive for anti-amphiphysin antibodies. Pulmonary biopsy led to the diagnosis of a small-cell lung carcinoma, which had been detected by computer tomography earlier. He received a 5-day-lasting IVIG treatment (0.4 g/kg/day) without any improvement. Intramuscular injections of botulinum toxin and symptomatic therapy with morphine, diazepam, and clonazepam reduced pain, but only chemo- and radiotherapy led to a significant pain relief and reduction of stiffness, so the patient could walk again. Eight months later he died due to a pulmonary embolism.

### 2.3. Patient 3

Patient 3 is a 68-year-old Caucasian male affected by the stiff limb syndrome (SLS), a SPS variant (the so-called Focal/Segmental SPS) where symptoms are confined to the limbs. At the age of 66 the patient started complaining about walking difficulties associated with pain in the inguinal region. Five years earlier he underwent to a right hip joint replacement followed by complete recovery. In the following months he developed stiffness and rigidity in both legs, predominantly in his right one, which worsened after walking for longer distances. Furthermore, annoying muscles cramps in his right thigh appeared. Three months later he started to present with recurrent vomiting, independently of eating. Investigations regarding intra- and extraperitoneal causes of vomiting, including central neurological disorders and metabolic diseases, were all normal as well as neurophysiological testing of the autonomic nervous system. Gut sensorimotor function was not further evaluated. Finally, the* Helicobacter pylori* breath test was positive and after antibiotic therapy a mild improvement of vomiting frequency occurred. In the following months the patient developed additionally dyspnoea after moderate physical stress and severe dysarthria, whereas cardiac-pulmonary investigations and brain and neck magnetic resonance imaging (MRI) were all unremarkable. This clinical deterioration led to a severe depression with diminished quality of life. EMG testing revealed variable hypertonic activity at rest in the anterior leg muscles. Laboratory investigations were positive for an extremely high anti-GAD-antibody titer (>2.000 KUI/l compared to a reference value of <10 KUI/l). Therapeutic attempts with low doses of baclofen, escitalopram, diazepam, and clonazepam had to be interrupted due to the patient's vomiting and important side effects such as tiredness and dizziness. The patient was treated with three sessions of IVIG (0.4 g/kg/day for 5 days) resulting only in very mild and short-lasting benefits; therefore, further sessions of IVIG were abandoned. In contrast, treatment with prednisone (60 mg/day, followed by tapering) induced a rapid and significant reduction of stiffness and dysarthria and complete remission of vomiting. Unfortunately, chronic corticosteroid treatment rapidly led to severe osteoporosis with vertebral fractures. Azathioprine (AZA) has therefore been recently added to the patient's therapy. However, only 25 mg/day of AZA seemed not to be well tolerated by the patient, as he started to complain about drowsiness and unusual weakness, and therefore he now takes it irregularly. If AZA is not tolerated, treatment with mycophenolate mofetil will be probably started.

A summary of the main characteristics of the three patients is shown in Table 1.

## 3. Discussion

We here report cases of three patients affected by classic SPS (Patient 1), paraneoplastic SPS (Patient 2), and Focal/Segmental SPS (Patient 3). All three patients were referred to the Department of Neurology due to muscular stiffness associated with muscle cramps and spasms. Symptoms differed in location and severity. In Patient 1, stiffness was mainly confined to the axial muscles, while in the other two patients confinement was to their lower limbs with unilateral predominance. Muscle spasms induced by external stimuli were an important cause of respiratory disability in Patient 1, while muscle cramps in legs were extremely painful in Patient 2, even requiring morphine treatment. Muscle cramps in Patient 3 were neither very painful nor disabling. The disease progression was highly variable, too, going from very fast clinical deterioration (within weeks) of Patient 2 to slowly but steadily progressive devolution (within years) of Patient 1, with the disease progression of Patient 3 somewhere in between (within months).

The first-line treatment for symptomatic management showed good results only in Patient 1, but only during the first years of therapy. Increased desensitization to treatment combined with disease progression made the continuous increasing of drug dose necessary, leading to important side effects, addiction, and low compliance. The symptomatic treatment in Patient 3 had to be interrupted several times because of adverse effects, even at low doses, while Patient 2 did not benefit from it at all. Also the response to IVIG, which is considered as the best second-line treatment for patients with severe or refractory SPS [6], was effective only in Patient 1. However, the response to IVIG has become less effective and fluctuating after some years. IVIG was without any benefit in Patient 2, while Patient 3 improved only lightly. The evidence supporting PE for SPS is less well established than for IVIG and its effectiveness is still uncertain [7]. Furthermore, although PE is considered as a safe and well-tolerated procedure [7], attempts with PE in Patient 1 had to be interrupted due to thrombotic events. May be treatment with rituximab, a B cell-depleting monoclonal antibody, could be an option in Patient 1. Rituximab is administered intravenously, usually twice in the beginning and then every half a year. This kind of drug administration indeed does not require a particular patient's adherence, even if Patient 1 frequently misses medical appointments. However, there are only a few randomized controlled trials of rituximab in SPS, without showing real efficacy [8]. Most data in the literature derive from case reports where in some of the described patients rituximab seemed to be a promising treatment option [9]. Nevertheless, single experiences are not meaningful and there is of course need for controlled studies. Unfortunately, conducting randomized studies in such a rare disease is very difficult and almost impossible. Finally, therapeutic decisions are additionally influenced by the type I DM of Patient 1, which is scarcely controlled, and by his reduced liver function. Also the recurrent vomiting of Patient 3, associated with early onset of therapeutic adverse effects, caused significant disruption especially to the first-line treatment strategies and their success with numerous therapies needing to be interrupted.

The clinical manifestation “vomiting” of Patient 3 is particularly interesting, as this uncommon feature has not been routinely described in SPS patients yet. To our knowledge only one case has been reported in the literature [10], where a SLS patient initially suffered from hiccup and vomiting only during summer months and then demonstrating more muscular symptoms. As the vomiting responded to diazepam, the authors hypothesized an underlying diaphragmatic spasm. In our patient, the pathogenic mechanism of vomiting is unknown, but as treatment with corticosteroids provoked its complete remission it can be speculated that the vomiting is indeed another symptom of SPS, as well as his progressive dysarthria, which has also significantly improved with prednisone. Paroxysmal autonomic dysfunctions and sphincter or brainstem involvement in SPS have been reported in the literature [11, 12], but without explicit mentioning of vomiting or dysarthria. Patient 3 might possibly develop a GAD-antibody associated cerebellar ataxia that, beyond SPS, is the most frequent presentation of anti-GAD-associated neurological disorders [8]. However, so far the patient has not developed a gait or limb ataxia. Another explanation of the patient's recurrent vomiting could be an involvement of the area postrema in SPS, which is located on the dorsal surface of the medulla oblongata and implicated in the central neurocircuitries of vomiting [13]. Some autoimmune conditions are associated with cyclic vomiting, especially the neuromyelitis optica (NMO). NMO is characterized by optic neuritis, transverse myelitis, and the presence of anti-aquaporin-4 antibodies in the serum. Its clinical spectrum has been recently expanded into NMO spectrum disorders including also intractable nausea, vomiting, or hiccups within the clinical manifestations [14]. Neuropathologic studies suggest that the area postrema may be a selective autoimmunity target of the disease process in NMO as aquaporin-4 is abundantly expressed in that area [15]. An extensive immunohistochemical study of the rat brainstem demonstrated numerous GAD67-expressing neurons in the area postrema and other regions of the brainstem [16]. May be the area postrema and brainstem are a target of anti-GAD-antibodies in Patient 3, which would explain his vomiting and dysarthria. In SPS, however, the isoform GAD65 is usually the main target for the anti-GAD-antibodies and not the isoform GAD67. Furthermore, the brain MRI of Patient 3 was completely normal without showing abnormalities in the brainstem, especially in the area postrema.

In summary, SPS is highly heterogeneous in clinical presentation, diagnostic findings, disease progression, and treatment response, but in most cases it is very disabling leading to a significant reduction of quality of life. The description of single case, especially of those with uncommon features, is important to improve the knowledge of that rare disease. In addition, multitask therapy of SPS is often unsatisfactory and not well tolerated. Finally, therapy becomes less effective during clinical progression and it is highly influenced by the patient's compliance and associated health problems. Therefore additional longer-lasting and better-tolerated therapeutic strategies are still needed.

## Figures and Tables

**Table 1:** Main characteristics of the three SPS patients.

Pt. nr./SPS form	Sex/age (yrs)	Age of onset (yrs)	Symptoms of onset	Distribution of pain and stiffness	Velocity of progression	Ab	EMG findings	Associated diseases/symptoms	SPS treatment
Patient 1/classic SPS	M/45	36	Pain, stiffness, and muscle cramps	Trunk (axial), followed by limbs (proximal) and face	Years	GAD (low titer)	Axonal sensory-motor polyneuropathy; CMUA at rest	Type I DM; hepatic steatosis; agoraphobia; depression	bzd, CBZ and gabapentin; IVIG

Patient 2/paraneoplastic SPS	M/73	72	Pruritus, severe pain, stiffness, and muscle cramps	Left foot, followed by both feet and both legs	Weeks	AMPH	Left peroneal nerve lesion; CMUA at rest	Small-cell lung carcinoma	bzd; morphine; chemo- and radiotherapy of tumor

Patient 3/SLS	M/68	66	Gait difficulty, pain, and stiffness (> after walking)	Legs (≫ right) (> proximal)	Months	GAD (very high titer)	Variable hypertonic activity	Recurrent vomiting; dysarthria; depression	Prednisone and AZA

Pt.: patient; nr.: number; SPS: stiff person syndrome; yrs: years; Ab: antibodies; EMG: electromyography; M: male; GAD: glutamic acid decarboxylase; CMUA: continuous muscular activity; DM: diabetes mellitus; bzd: benzodiazepines; CBZ: carbamazepine; IVIG: intravenous immunoglobulin; AMPH: amphiphysin; SLS: stiff limb syndrome; >: especially; AZA: azathioprine.

## List of Abbreviations

AZA: Azathioprine
CMUA: Continuous motor unit activity
DM: Diabetes mellitus
EMG: Electromyography
GABA: Gamma-aminobutyric acid
GAD: Glutamic acid decarboxylase
IVIG: Intravenous immunoglobulin
MRI: Magnetic resonance imaging
NMO: Neuromyelitis optica
PE: Plasma exchange
SLS: Stiff limb syndrome
SPS: Stiff person syndrome.
